# The comparison of nailfold videocapillaroscopy findings between anti-melanoma differentiation-associated gene 5 antibody and anti-aminoacyl tRNA synthetase antibody in patients with dermatomyositis complicated by interstitial lung disease

**DOI:** 10.1038/s41598-020-72752-7

**Published:** 2020-09-24

**Authors:** Reiko Wakura, Shogo Matsuda, Takuya Kotani, Takeshi Shoda, Tohru Takeuchi

**Affiliations:** 1 Department of Internal Medicine IV, Division of Rheumatology, Osaka Medical College, 2-7 Daigaku-Machi, Takatsuki, Osaka 569-8686 Japan; 2 Department of Rheumatology, Internal Medicine, Yodogawa Christian Hospital, Osaka, Japan

**Keywords:** Immunology, Rheumatology, Signs and symptoms

## Abstract

Dermatomyositis (DM) is frequently complicated by interstitial lung disease (ILD), which increases mortality. This study aims to elucidate the clinical significance of nailfold videocapillaroscopy (NVC) on assessing the disease activity and prognosis of DM-ILD. We compared the NVC findings between anti-melanoma differentiation-associated gene 5 (anti-MDA5) antibody-positive and anti-aminoacyl tRNA synthetase (anti-ARS) antibody-positive patients, the survival and ILD-related death groups, and examined the association of NVC findings with prognostic factors of DM-ILD. The median scores of microhemorrhage and capillary disorganization in the anti-MDA5 antibody-positive group were significantly higher than those in the anti-ARS antibody-positive group (*P* = 0.012 and 0.044, respectively). In contrast, the median scores of tortuous capillaries in the anti-ARS antibody-positive group were significantly higher than those in the anti-MDA5 antibody-positive group (*P* = 0.002). The median scores of microhemorrhage was significantly higher in the ILD-related death group than the survival group (*P* = 0.02). The scores of microhemorrhage, capillary disorganization, and neoangiogenesis correlated with known poor prognosis factors of DM-ILD. Additionally, the scores of microhemorrhage and capillary loss correlated significantly with the total fibrosis scores of chest high-resolution computed tomography. These findings suggest that NVC is a useful tool for assessing the disease activity and prognosis of DM-ILD.

## Introduction

Polymyositis/dermatomyositis (PM/DM) is one of the autoimmune-inflammatory diseases and commonly causes muscle weakness mainly of the trunk, proximal extremities, neck, and pharynx^1,2^. DM is characterized by the presence of skin rashes called heliotrope rash and Gottron’s sign^3,4^. DM is frequently complicated by interstitial lung disease (ILD), which causes increased morbidity and mortality^5^. Currently, myositis-specific autoantibodies, serum ferritin levels, serum Krebs von den lungen-6 (KL-6) levels, serum C-reactive protein (CRP) levels, alveolar-arterial oxygen difference (AaDO_2_), and chest high-resolution computed tomography (HRCT) findings were reported to be related with the diagnosis and severity in DM-ILD patients^6–11^. However, these poor prognostic factors were insufficient to evaluate the diagnosis and severity. Therefore, new tools are needed to further improve the accuracy of diagnosis and severity assessment.

Nailfold videocapillaroscopy (NVC) is a noninvasive, safe, and real-time method of assessing microvascular abnormalities in the nailfold^12,13^, and is useful for the diagnosis and disease severity in connective tissue diseases, especially systemic sclerosis (SSc). In SSc, vasculopathy is recognized from the early stages of the disease, and microvascular abnormalities such as giant capillaries, microhemorrhages, and capillary loss, are shown in NVC^14^. The presence of vasculopathy in NVC findings is included in the classification criteria for SSc, and NVC findings reflect the clinical stages of SSc^15,16^.

Recently, the relationship between NVC findings and clinical features has been examined in connective tissue diseases other than SSc. Abnormal NVC findings are also seen in DM patients^17^. Myositis activity has been reported to correlate significantly with loss of capillaries on NVC findings in DM patients^18^. Kubo et al. reported a significant correlation between NVC findings and perivascular lymphocyte infiltrations in skin biopsies of DM patients^19^. However, NVC findings in DM-ILD has not been elucidated. Therefore, in this study, we investigated the relationship between NVC findings and clinical features in DM-ILD patients and evaluated the significance of NVC findings between anti-melanoma differentiation-associated gene 5 antibody and anti-aminoacyl tRNA synthetase antibody.

## Methods

### Patients

We examined patients who were admitted to Osaka Medical College Hospital from May 2015 to April 2018 in this retrospective study. They were diagnosed as having DM or clinically amyopathic DM based on the criteria of Bohan and Peter^1,2^ or Sontheimer and Gerami^3,4^ et al. Patients with other connective tissue diseases and malignancies were excluded. ILD was diagnosed with chest HRCT. Acute/subacute interstitial pneumonia (A/SIP) was defined as ILD in which the respiratory condition, laboratory findings, arterial blood gas findings, chest HRCT images, and pulmonary function test findings rapidly aggravated within 3 months^20^. Chronic interstitial pneumonia (CIP) did not fulfill the definition of A/SIP. Clinical data were obtained from the patients’ medical records on admission. This study was conducted in accordance with the Declaration of Helsinki and its amendments and was approved by Osaka Medical College and the Faculties of Medicine Ethics Committee (approval no. 1598). Informed consent was obtained from each patient.

### Treatment for patients with DM-ILD

Prednisolone (PDN) (0.5–1.0 mg/kg/day) was administered in 22 of the 27 patients. Cyclosporine (CSA) or tacrolimus (TAC) was used as combination treatment according to the physician’s decision. CSA was started at 4 mg/kg/day once a day before breakfast, and the concentration at 2 h after administration was adjusted to 1,500 ng/mL or above. TAC was started at 0.1 mg/kg/day twice a day before breakfast and dinner, and the trough was adjusted to 5–15 ng/mL^21^. Whether additional treatments such as methylprednisolone pulse therapy (MPDN), intravenous pulse cyclophosphamide, or intravenous immunoglobulin were administered was determined by the physician depending on each patient’s condition.

### Measurement of clinical signs and laboratory parameters

The clinical signs, including cutaneous ulcerations, mechanic’s hands, Raynaud’s phenomenon, Gottron’s sign/papules, palmar papules, and arthritis, were evaluated. The laboratory parameters measured were albumin, creatine kinase, aldolase, lactic acid dehydrogenase, CRP, KL-6, surfactant protein-D, and ferritin. Anti-MDA5 antibody and anti-ARS antibody were examined by ELISA (MESACUP; MBL, Nagoya, Japan) and blot assay (Myositis Profile Euroline Blot test kit; EUROIMMUN, Lübeck, Germany), respectively^21^.

### Arterial blood gas analysis and pulmonary function test

Arterial blood gas analysis including PaO_2_, PaCO_2_, and AaDO_2_ was conducted on admission. Respiratory function was measured by spirometry (SYSTEM21; Minato Medical Science, Osaka, Japan). Vital capacity was determined by the N2 washout method, and diffusion capacity of the lung for carbon monoxide was determined by the single-breath method^22–24^. Respiratory function test results are expressed as percentages of the predicted value.

### HRCT scoring

HRCT was performed using a 64-detector row CT Aquilon multiscanner (Toshiba Medical Systems Corporation, Tokyo, Japan). Slice thickness was 1.0–1.5 mm every 10 mm, with the scan area including the entire lung. All patients underwent chest HRCT prior to treatment, and images were reviewed independently by 3 observers (SM, TK, and TS) blinded to the patients’ clinical information. Inter-observer disagreements were resolved by consensus. Ground-glass opacity (GGO) and fibrosis were both scored to assess HRCT findings as described previously^25^. The lobes of each patient were scored by the same observers, and the average of the three values was used. The scores obtained were summed as the total CT score. Because the right middle lobe GGO score is strongly related to the poor prognosis of DM-ILD^26^, it was considered as a poor prognostic factor.

### Nailfold videocapillaroscopy

NVC was performed using a Dino-lite capillaroscopy device (with Dinocapture 2.0 windows software) at 200 × magnification. Patients were acclimated to a room temperature of 20–23 °C at least 15 min before the exam. Immersion oil was placed on the nailfold beds to improve the image resolution. All images were analyzed by two independent rheumatologists (RW and TS) who were blinded to the patients’ clinical diagnosis and disease severity. We took two images of the middle of the nailfold for all fingers of both hands excluding the thumbs.

**Figure 1:**
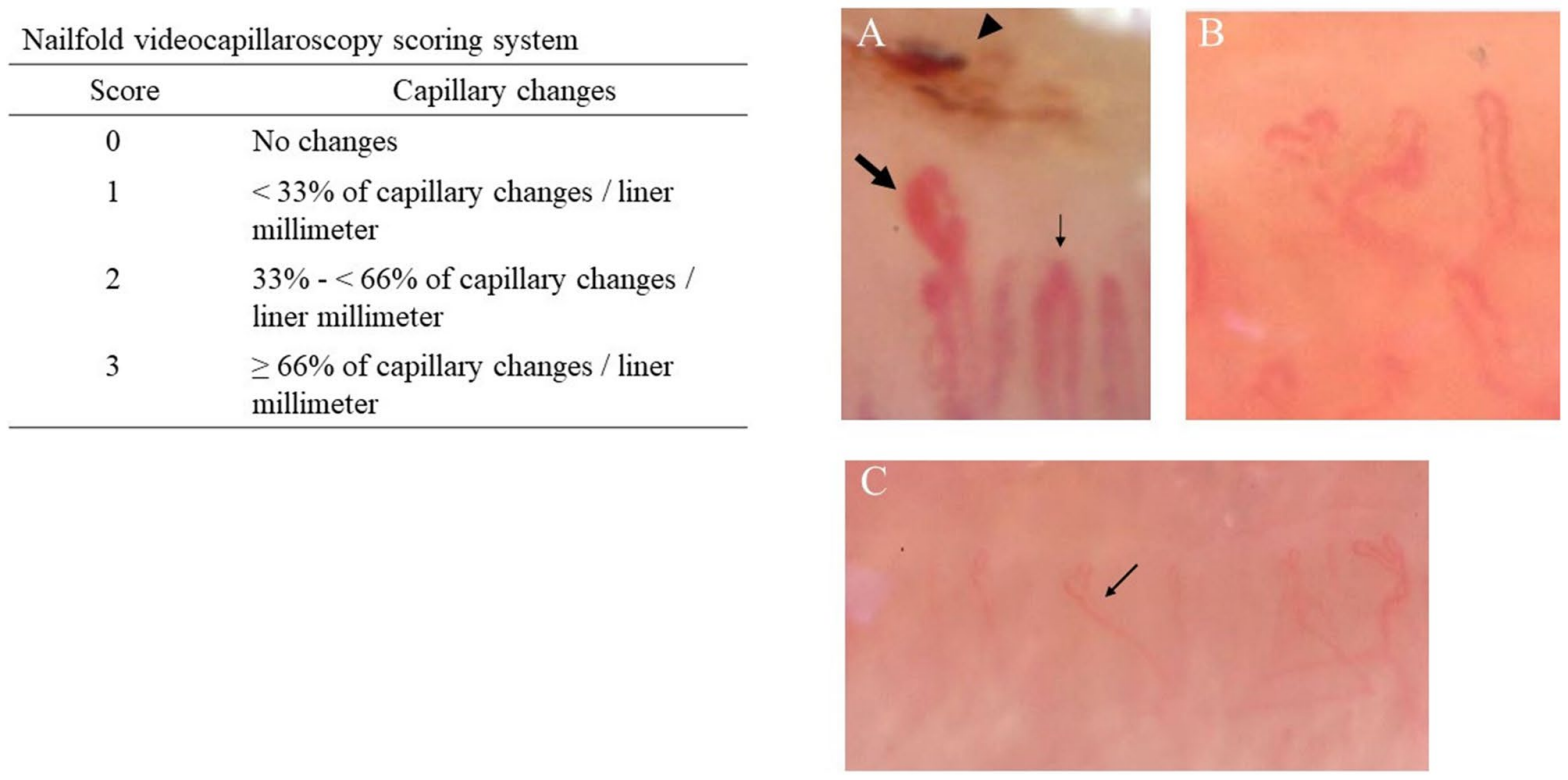
Nailfold videocapillaroscopy scoring system based on Cutolo et al. (26). (**A**) Microhemorrhage (arrowhead), giant capillary (wide arrow), enlarged capillary (narrow arrow). (**B**) Neoangiogenesis. (**C**) Loss of capillary and capillary disorganization (arrow). Magnification 200× .

A semiquantitative rating scale to score NVC findings was adopted as follows according to previous studies^27,28^; 0 = no changes; 1 = less than 33% of capillary alterations/reduction, 2 = 33–66% of capillary alterations/reduction, 3 = more than 66% of capillary alterations/reduction, per linear millimeter. The mean score values of all the capillaroscopic parameters were calculated. The following hallmark parameters were counted: enlarged capillaries, giant capillaries, microhemorrhages, loss of capillaries, disorganization, neoangiogenesis (bushy and bizarre capillaries), and cross and tortuous capillaries (Fig. 1).

### Statistical analysis

Data are presented as the median (interquartile range). Fisher’s exact test was used when appropriate, and the Mann–Whitney U-test was used for the comparison of median values. Correlations were evaluated using Spearman’s correlation coefficients. A *P* value of < 0.05 was considered to indicate statistical significance. The data were analyzed using JMP version 14.0 (SAS Institute Inc., Cary, NC, USA).

## Results

### Patient profiles

The profiles of the 27- DM-ILD patients examined, of whom 24 survived and 3 died due to ILD, are shown in Table 1. The median patient age was 57 (50–67) years, and 24 (88.9%) patients were women. Fourteen patients (51.9%) had clinically amyopathic DM, 13 (48.1%) had DM, and 13 (48.1%) had A/SIP. The median time from the appearance of respiratory symptoms to treatment initiation (disease duration) was 13.1 (2–100) weeks. Ten patients (37%) were anti-MDA5 antibody positive and 17 (63%) DM-ILD patients were anti-ARS antibody positive. The frequency of CIP was significantly higher in patients with anti-ARS antibody positive (76.5%) than that in patients with anti-MDA5 antibody positive (10%) (*P* = 0.0013).

**Table1:** Demographic, clinical, and serological features of 27 patients with DM-ILD.

Features	Values
Age, years	57 (50–67)
Female, n (%)	24 (88.9)
**Disease type, n (%)**	
CADM	14 (51.9)
A/SIP, n (%)	13 (48.1)
**Symptoms, %**	
Mechanic’s hands	14 (51.9)
Raynaud’s phenomenon	4 (14.8)
Gottron's sign	15 (55.6)
Palmar papules	6 (22.2)
Cutaneous ulcerations	3 (11.1)
Arthritis	6 (22.2)
Disease duration, weeks	13.1 (2–100)^a^
Positive anti-MDA5Ab, n (%)	10 (37.0)
Positive anti-ARS Ab, n (%)	17 (63.0)
**Laboratory findings**	
Alb, mg/dl	3.7 (3.1–4)
CK, IU/l	105 (56–203)
ALD, IU/l	5.9 (3.7–8.4)^b^
LDH, IU/l	233 (190–414)
CRP, mg/dl	0.12 (0.04–1.48)
KL-6, U/ml	772 (480–1,153)
SP-D, ng/mL	112 (36.5–222.5)^c^
Ferritin, ng/ml	159.4 (90.8–634.7)^d^
AaDO_2_, mmHg	16.9 (5.2–40.1)^e^
**PFTs**	
%FVC, %	87.9 (72.5–93.8)^f^
%Dlco, %	56.5 (46.6–66.6)^g^
**Chest HRCT scores**	
Right middle lobe GGO score	1.3 (1–2)
Total GGO score	7.3 (5–11.3)
Total fibrosis score	3.3 (2–5)
**Treatments**	
PDN (n = 22), mg/day	45 (30–56)
CSA (n = 8), mg/day	200 (131–250)
TAC (n = 11), mg/day	4 (3–4)
MPDN, n (%)	5 (18.5)
Total IVCY (n = 12), mg	4,750 (3,000–6,500)
IVIg, n (%)	8 (29.6)
**Prognosis, n (%)**	
Alive	24 (88.9)
Dead	3 (11.1)

DM, dermatomyositis; ILD, interstitial lung disease; CADM, clinical amyopathic dermatomyositis; A/SIP, acute/subacute interstitial pneumonia; MDA5, anti-melanoma differentiation-associated gene 5; Ab, antibody; ARS, aminoacyl-tRNA synthetase; Alb, albumin; CK, creatine kinase; ALD, aldorase; LDH, lactate dehydrogenase; KL-6, Krebs von den Lungen-6; SP-D, surfactant protein-D; AaDO_2_, alveolar-arterial oxygen difference; FVC, forced vital capacity; DLco, diffusion capacity of the lung for carbon monoxide; GGO, ground-glass opacity; PDN, prednisolone; CSA, cyclosporine; TAC, tacrolimus; MPDN, methylprednisolone pulse therapy; IVCY, intravenous pulse cyclophosphamide; IVIg, intravenous immunoglobulin; Dead, dead due to ILD. The laboratory markers are presented as the median (interquartile range).

^a^Number of subject, n = 23.

^b^Number of subject, n = 24.

^c^Number of subject, n = 25.

^d^Number of subject, n = 24.

^e^Number of subject, n = 21.

^f^Number of subject, n = 9.

^g^Number of subject, n = 8.

Other laboratory findings, such as clinical symptoms, AaDO_2_, respiratory function test results, and chest HRCT scoring results are listed in Table 1. PDN was used in 22 patients, and the median dose was 45 (30–56) mg/day. CSA were used in 8 patients and the median doses were 200 (131–250) mg/day. TAC were used in 11 patients and the median doses were 4 (3–4) mg/day. MPDN pulse and intravenous immunoglobulin therapy were used in 5 and 8 patients, respectively. Intravenous pulse cyclophosphamide was used in 12 patients, and the median total dose was 4,750 (3,000–6,500) mg. All patients who died were anti-MDA5 antibody positive.

### Comparison of NVC findings between anti-MDA5 antibody-positive and anti-ARS antibody-positive patients

**Figure 2:**
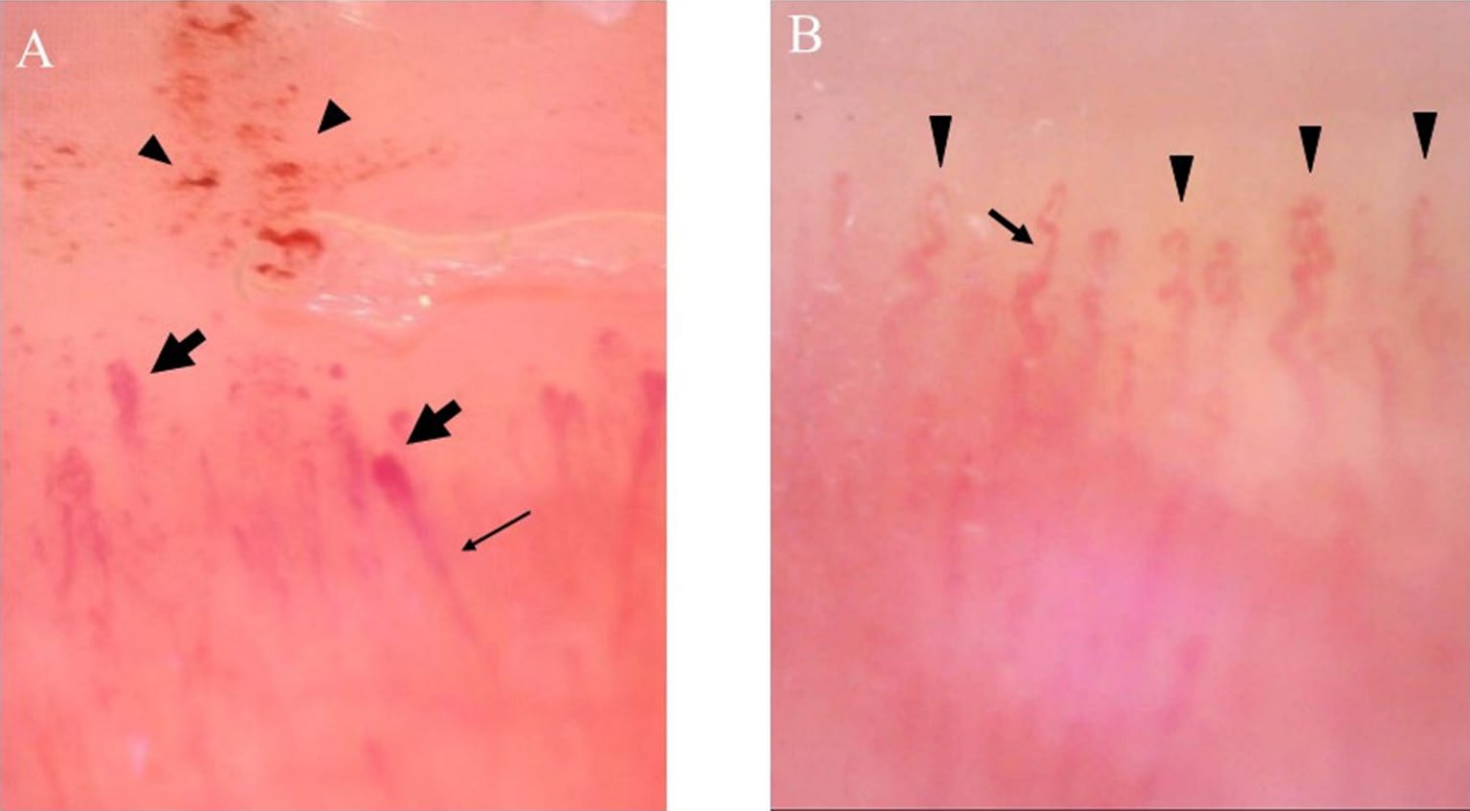
Nailfold videocapillaroscopy findings of DM-ILD. (**A**) Typical findings in a case of anti-MDA5-Ab-positive DM-ILD: microhemorrhages (arrowhead), enlarged capillary (wide arrow), and capillary disorganization (arrow). (**B**) Typical findings in a case of anti-ARS-Ab-positive DM-ILD: a cross capillary (arrow) and tortuous capillary (arrowhead). Magnification 200× .

NVC findings were assessed before treatments in all patients. The scores of NVC findings in all DM-ILD patients are shown in Supplementary Table S1, and those between the anti-MDA5 antibody-positive and anti-ARS antibody-positive patients are shown in Table 2. The median scores of microhemorrhage and capillary disorganization were significantly higher in the anti-MDA5-Ab-positive group (0.71 [0.36–1.34] and 0.84 [0.44–1.28], respectively) than those in the anti-ARS-Ab-positive group (0.25 [0–0.5] and 0.33 [0–0.75], respectively) (*P* = 0.012 and 0.044, respectively). In contrast, the median score of tortuous capillaries was significantly higher in the anti-ARS-Ab-positive group (1.33 [1.06–1.65]) than that in the anti-MDA5-Ab-positive group (0.55 [0.09–1.03]) (*P* = 0.002). Typical NVC findings of an anti-MDA5-Ab-positive and an anti-ARS-Ab-positive DM-ILD case are shown in Fig. 2.

**Table 2:** Comparison of nailfold videocapillaroscopy findings between anti-MDA5 antibody positive and anti-ARS antibody positive DM-ILD patients.

Findings	Associated antibody		*P*
	Anti-MDA5-Ab (+) N = 10	Anti-ARS-Ab (+) N = 17	
Enlarged capillary	1.2 (0.33–2)	1.5 (0.45–2.38)	0.530
Giant capillary	0 (0–0.78)	0 (0–0.18)	0.369
Microhemorrhage	0.71 (0.36–1.34)	0.25 (0–0.5)	0.012*
Capillary loss	1.56 (0.68–1.76)	1.44 (0.63–1.78)	0.841
Capillary disorganization	0.84 (0.44–1.28)	0.33 (0–0.75)	0.044*
Neoangiogenesis	0.38 (0–0.87)	0 (0–0.38)	0.136
Bushy capillary	0.19 (0–0.37)	0 (0–0.2)	0.139
Bizzare capillary	0.25 (0–0.5)	0 (0–0.23)	0.214
Cross capillary	0.69 (0.5–1.06)	0.75 (0.5–1.16)	0.800
Tortuous capillary	0.55 (0.09–1.03)	1.33 (1.06–1.65)	0.002**

MDA5, anti-melanoma differentiation-associated gene 5; ARS, aminoacyl-tRNA synthetase; DM, dermatomyositis; ILD, interstitial lung disease.

The laboratory markers are presented as the median (interquartile range). The *P*-values were estimated using Wilcoxon rank sum test. *P < 0.05, **P < 0.01.

### Comparison of NVC findings between the survival and death due to ILD groups

NVC findings between the survival group and death due to ILD group can be compared in Table 3. The median microhemorrhage score was significantly higher in the death due to ILD group (0.8 [0.75–2.5]) than that in the survival group (0.33 [0.05–0.5]) (*P* = 0.02). Also, the median neoangiogenesis score was significantly higher in the death due to ILD group (0.83 [0.5–1.6]) than that in the survival group (0 [0–0.38]) (*P* = 0.02). There were no significant differences in the other findings.

**Table 3:** Comparison of nailfold videocapillaroscopy findings between dead groups and alive groups in DM-ILD.

Findings	Prognosis		*P*
	Dead due to ILD N = 3	Alive N = 24	
Enlarged capillary	1.75 (0.8–2)	1.2 (0.35–2.13)	0.79
Giant capillary	0.75 (0–1.33)	0 (0–0.19)	0.10
Microhemorrhage	0.8 (0.75–2.5)	0.33 (0.05–0.5)	0.02*
Capillary loss	1.8 (1.5–1.83)	1.41(0.62–1.75)	0.15
Capillary disorganization	1.2 (1–1.5)	0.38 (0.10–0.79)	0.04*
Neoangiogenesis	0.83 (0.5–1.6)	0 (0–0.38)	0.02*
Bushy capillary	0.33 (0.25–0.8)	0 (0–0.22)	0.03*
Bizzare capillary	0.5 (0.25–0.8)	0 (0–0.25)	0.03*
Cross capillary	1.0 (0.5–1.0)	0.69 (0.5–1.18)	1.00
Tortuous capillary	0.6 (0.17–1)	1.2 (0.53–1.5)	0.16

DM, dermatomyositis; ILD, interstitial lung disease.

The laboratory markers are presented as the median (interquartile range). The *P*-values were estimated using Wilcoxon rank sum test. *P < 0.05.

As shown in Supplementary Table S2, we also compared the differences in NVC findings of anti-MDA5 antibody-positive DM-ILD between survivors (N = 7) and dead patients (N = 3). The median scores for microhemorrhage, capillary loss, capillary disorganization, and neoangiogenesis tended to be higher in the death due to ILD group than those in the survival group (*P* < 0.2). However, there was no statistical significance between the two groups.

### Correlation between NVC findings and disease activity indicators of DM-ILD

The correlations between NVC findings and disease activity indicators of DM-ILD are shown in Table 4. The microhemorrhage score correlated significantly with the serum levels of CRP (*R* = 0.48), ferritin (*R* = 0.41), AaDO2 (*R* = 0.45), total GGO scores (*R* = 0.39), and total fibrosis scores (*R* = 0.40). The score of giant capillaries correlated significantly with the serum levels of ferritin (*R* = 0.45) and the right middle lobe GGO scores (*R* = 0.58). The score of capillary disorganization correlated significantly with ferritin (*R* = 0.51) and with AaDO_2_ (*R* = 0.66). The neoangiogenesis score correlated significantly with the serum levels of ferritin (*R* = 0.46), AaDO_2_ (*R* = 0.50), and the total GGO scores (*R* = 0.65). The score of bushy capillaries correlated significantly with the serum levels of ferritin (*R* = 0.41), AaDO_2_ (*R* = 0.60), and the total GGO scores (*R* = 0.53). Also, the scores of capillary loss correlated significantly with the total fibrosis scores (*R* = 0.73).

**Table 4:** Correlation of nailfold videocapillaroscopy findings with disease activity factors of DM-ILD.

Findings	Age	Disease duration	Laboratory findings					DL	Pulmonary function tests		Chest HRCT scoring		
			CK	LD	CRP	KL-6	Ferritin	AaDO_2_	%FVC	%Dlco	Right middle lobe GGO score	Total GGO score	Total fibrosis score
Enlarged capillary	0.52**	0.11	− 0.25	− 0.17	− 0.06	0.13	0.16	0.19	0.22	0.33	0.37	0.03	0.35
Giant capillary	0.29	− 0.14	− 0.08	0.05	0.27	0.20	0.45*	0.30	0.51	0.52	0.58**	0.35	0.46*
Microhemorrhages	0.17	− 0.46*	0.19	0.54**	0.48*	0.35	0.41*	0.45*	− 0.03	− 0.20	0.29	0.39*	0.40*
Capillary loss	0.23	0.16	− 0.23	− 0.29	0.005	0.17	0.03	0.03	− 0.13	0.36	0.44*	0.31	0.73**
Capillary disorganization	0.51**	− 0.49*	0.02	0.30	0.30	0.001	0.51*	0.66**	0.54	0.71*	0.21	0.19	0.23
Neoangiogenesis	0.40*	− 0.22	0.12	0.18	0.20	0.03	0.46*	0.50*	− 0.09	0.27	0.35	0.65**	0.44*
Bushy capillary	0.27	− 0.32	0.25	0.22	0.18	0.04	0.41*	0.60**	− 0.37	0.41	0.23	0.53**	0.32
Bizzare capillary	0.51**	− 0.15	0.07	0.15	0.19	− 0.08	0.40	0.40	0.31	0.27	0.32	0.48*	0.39*
Cross capillary	0.41*	0.14	− 0.01	− 0.01	0.16	− 0.14	0.33	0.03	0.08	− 0.40	0.30	0.25	− 0.01
Tortuous capillary	0.25	0.58**	− 0.50*	− 0.43*	− 0.35	0.08	− 0.22	− 0.22	− 0.28	0.17	− 0.01	− 0.29	0.07

DM, dermatomyositis; ILD, interstitial lung disease; DL, *Diffusing capacity* of the lung; HRCT, high-resolution computed tomography; CK, creatine kinase; LD, lactate dehydrogenase; CRP, C-reactive protein; KL-6, Krebs von den Lungen-6; AaDO_2_, alveolar-arterial oxygen difference; FVC, forced vital capacity; DLco, diffusion capacity of the lung for carbon monoxide; GGO, ground-glass opacity.

Correlations were evaluated using Spearman’s rank correlation coefficient. *P < 0.05, **P < 0.01.

## Discussion

In this study, we investigated the relationship between NVC findings and clinical features in DM-ILD patients. A high microhemorrhage score was observed in the NVC findings in the anti-MDA5 antibody-positive patients and the death due to ILD group, and correlated with the poor prognostic factors of DM-ILD. A high tortuous capillary score was observed in anti-ARS antibody-positive patients. The score of capillary loss was associated with the extent of lung fibrosis. The NVC findings were associated with myositis-specific autoantibodies, severity, and the extent of lung fibrosis in DM-ILD patients.

Microhemorrhages are frequently observed in NVC findings in patients with DM, probably due to the microvasculopathy associated with vascular endothelial injury^29^. Mugii et al. reported that microhemorrhage in NVC findings and are correlated with skin disease activity and considered to be reversible because it decreases after immunosuppressive therapy in DM patients^18,30^. Fiorentino et al. reported that the skin ulcers of anti-MDA5 antibody-positive DM patients had spread to the nail side, inflammatory cell infiltrations were observed around the capillaries in the biopsy tissue, and they exhibited severe vasculopathy^31^. Also, Okiyama et al. reported that vascular injury in skin biopsy was observed more often in the anti-MDA5 antibody-positive group than in the anti-ARS antibody-positive groups and anti-transcriptional intermediary factor-1 antibody-positive groups^32^. These reports support the results of the present study, which showed that anti-MDA5 antibody-positive DM patients had high scores of microhemorrhage and capillary disorganization in the NVC findings. In the present study, the poor prognostic factors of DM-ILD, such as serum ferritin, CRP, and KL-6 levels, and chest HRCT findings, correlated with the scores of microhemorrhage and capillary disorganization in the NVC findings. Also, the scores of microhemorrhage were significantly higher in the death due to ILD group than that in the survival group. In our study, all dead patients were anti-MDA5 antibody-positive, which indicated severe vasculopathy in skin compared to anti-ARS antibody-positive groups^33^. Therefore, the score of hemorrhage, which reflects the severity of vasculopathy, in dead patients are significantly higher than those in the survival patients. Therefore, microhemorrhages in NVC findings are valuable markers for the evaluation of disease activity and prognosis in DM-ILD.

In anti-MDA5 antibody-positive DM-ILD, the median scores of microhemorrhage, tended to be higher in the death due to ILD groups than those in the survival group. This result might suggest that the dead group in anti-MDA5 antibody positive DM-ILD showed severe vasculopathy compared to the survival group. However, there was no significance between two groups due to limitation of small sample size.

Tortuous capillaries in NVC findings represent a pathology of neoangiogenesis resulting from chronic capillary injury^29^. This study showed that the positive correlation between the tortuous capillaries score in the NVC findings and the disease duration of DM-ILD patients. Also, the frequency of CIP was significantly higher in patients with anti-ARS antibody than that in patients with anti-MDA5 antibody. Therefore, patients with anti-ARS antibody had higher scores of tortuous capillaries compared to patients with anti-MDA5 antibody. Tortuous capillaries reflecting the neoangiogenesis could be valuable marker for the evaluation of chronicity and regeneration in DM-ILD.

Capillary loss in NVC findings is observed in the late phase in SSc patients and related to the skin fibrosis progression^16,34^. In the present study, there was a significant positive correlation between the score of capillary loss and the total fibrosis score on chest HRCT findings. A high total fibrosis score in the chest HRCT findings of DM-ILD patients expresses the state of ILD progression and the chronic phase after structural modification of the lung^25^. Thus, a high capillary loss score in the NVC findings is considered to represent the advanced and chronic stage of DM-ILD.

To our knowledge, the relationship between pulmonary pathology and NVC findings has not been examined in DM-ILD patients. A pulmonary biopsy was not obtained from all patients in this study and could not be compared with the NVC findings. However, in DM cases, the state of the capillaries in muscle and skin biopsies has been reported to be associated with NVC findings^19,35^, and microvasculopathy in NVC findings may be associated with lung lesions in DM-ILD patients. This is a retrospective study conducted in a single center with small number cases. To further clarify the association of NVC findings with the pathology of DM-ILD patients, it will be necessary to accumulate data by prospective analysis in a multi-center study.

## Conclusions

In conclusion, we investigated the relationship between NVC findings and clinical features in DM-ILD patients. The NVC findings were associated with myositis-specific autoantibodies, severity, and the extent of lung fibrosis in DM-ILD patients. NVC is a powerful tool for the prediction of disease activity and prognosis of DM-ILD.

## Supplementary information

Supplementary file1
