# Supplementary material for: The comparison of nailfold videocapillaroscopy findings between anti-melanoma differentiation-associated gene 5 antibody and anti-aminoacyl tRNA synthetase antibody in patients with dermatomyositis complicated by interstitial lung disease
Source: Sci Rep. 2020 Sep 24;10:15692. doi: 10.1038/s41598-020-72752-7 (PMC7518258; doi:10.1038/s41598-020-72752-7)
Supplement: Supplementary file 1 — Supplementary file1 [file 41598_2020_72752_MOESM1_ESM.docx]

Supplementary Information for:

**The Comparison of Nailfold Videocapillaroscopy Findings between Anti-melanoma Differentiation-associated Gene 5 Antibody and Anti-aminoacyl tRNA synthetase Antibody in Patients with Dermatomyositis Complicated by Interstitial Lung Disease**

Reiko Wakura^1^, Shogo Matsuda^1^, Takuya Kotani^1*^, Takeshi Shoda^2^, and Tohru Takeuchi^1^

Reiko Wakura and Shogo Matsuda contributed equally to this study.

^1^Department of Internal Medicine IV, Division of Rheumatology, Osaka Medical College, Takatsuki, Osaka, Japan

^2^Department of Rheumatology, Internal Medicine, Yodogawa Christian Hospital, Osaka, Japan

**Correspondence and reprint requests to** Takuya Kotani, MD, PhD

Department of Internal Medicine IV, Division of Rheumatology, Osaka Medical College, 2-7 Daigaku-Machi, Takatsuki, Osaka 569-8686, Japan

Tel.: +81-72-683-1221; Fax: +81-72-683-1801

E-mail: in1242@osaka-med.ac.jp

This supplement contains:

Supplementary Table S1

**Supplementary Table S1. The scores of nailfold videocapillaroscopy findings in DM-ILD patients.**

| Findings | Score |
| --- | --- |
| Enlarged capillary | 1.4 (0.4-2.1) |
| Giant capillary | 0 (0-0.25) |
| Microhemorrhage | 0.38 (0.19-0.67) |
| Loss of capillary | 1.5 (0.67-1.8) |
| Capillary disorganization | 0.5 (0.2-1) |
| Neoangiogenesis | 0.13 (0-0.5) |
| Bushy capillary | 0 (0-0.33) |
| Bizzare capillary | 0 (0-0.38) |
| Cross capillary | 0.69 (0.5-1.1) |
| Tortuous capillary | 1.1 (0.5-1.5) |

Abbreviations: DM, dermatomyositis; ILD, interstitial lung disease. The laboratory markers are presented as the median (interquartile range).

Supplementary Table S2

**Supplementary Table S2. Comparison of nailfold videocapillaroscopy findings between dead groups and alive groups in anti-MDA5 antibody positive DM-ILD patients.**

| Findings | Prognosis | |  |
| --- | --- | --- | --- |
|  | Dead due to ILD  N=3 | Alive  N=7 | *P* |
| Enlarged capillary | 1.8 (0.8-2) | 1 (0.31-2) | 0.57 |
| Giant capillary | 0.75 (0-1.33) | 0 (0-0.63) | 0.25 |
| Microhemorrhage | 0.8 (0.75-2.5) | 0.4 (0.31-1) | 0.17 |
| Capillary loss | 1.8 (1.5-1.83) | 0.88 (0.31-1.67) | 0.11 |
| Capillary disorganization | 1.2 (1-1.5) | 0.6 (0.25-1) | 0.14 |
| Neoangiogenesis | 0.83 (0.5-1.6) | 0.25 (0-0.38) | 0.06 |
| Bushy capillary | 0.33 (0.25-0.8) | 0 (0-0.25) | 0.08 |
| Bizzare capillary | 0.5 (0.25-0.8) | 0 (0-0.38) | 0.097 |
| Cross capillary | 1 (0.5-1) | 0.69 (0.5-1.25) | 0.91 |
| Tortuous capillary | 0.6 (0.17-1) | 0.5 (0-1.13) | 0.73 |

Abbreviations: MDA5, anti-melanoma differentiation-associated gene 5; DM, dermatomyositis; ILD, interstitial lung disease. The laboratory markers are presented as the median (interquartile range). The P-values were estimated using Wilcoxon rank sum test. *P <0.05.
